# The influenza virus sentinel surveillance: results of system evaluation in Mozambique, 2016-2021

**DOI:** 10.11604/pamj.2025.50.80.41994

**Published:** 2025-03-19

**Authors:** Samanta Djaló, Almiro Tivane, Neuza Nguenha, Nilsa Nascimento, Áuria Banze, Érika Rossetto, Cynthia Semá

**Affiliations:** 1 Mozambique Field Epidemiology and Training Program, Maputo, Mozambique,; 2 National Health Institute, Maputo, Mozambique,; 3 Centers for Disease Control and Prevention of Mozambique, Maputo, Mozambique

**Keywords:** Human influenza, Mozambique, pandemic, sentinel surveillance

## Abstract

The 2009 influenza pandemic has caused health challenges around the world. Mozambique has faced challenges in implementing surveillance systems, which are obstacles to the timely detection of outbreaks and epidemics. It is intended to evaluate the performance of the influenza sentinel surveillance system between 2016 and 2021. A descriptive-cross-sectional evaluation of the influenza sentinel surveillance system was conducted at the sentinel posts in Maputo. The sample was calculated, and a simple random sampling technique was used to select the 372 patient record forms. Microsoft Excel and Tableau were used for frequency calculations. Based on the Centers for Disease Control - 2001 script, data quality, stability, sensitivity, representativeness, timeliness, and positive predictive value were evaluated. 28.0% (1,305/4,660) of the analyzed samples had positive results, and 56.1% (2,617/4,660) were male. The system obtained data completeness and consistency of 69.9% (3,260/4,660) and 68% (355/372), respectively. It obtained a sensitivity of 77.5% (842/1,086) in 2017, a representative in 98.4% 1,285/1,305 of the neighborhoods, the opportunity of 50.4% (2,349/4,660), and a positive predictive value of 31.4% (410/1,305). The system proved to be useful, providing reliable data on influenza viral circulation. Continuous influenza monitoring would promote prevention interventions in the most vulnerable groups.

## Introduction

In recent years, acute viral respiratory infections have caused pandemics, epidemics and irregular outbreaks whose estimated 646,000 global deaths, 90% of which are attributable to children under 5 years of age [1,2]. In 1918 and 2009, the influenza A (H1N1) pdm09 pandemic was responsible for about 50 million and 18,000 deaths respectively [3,4]. African countries have faced several challenges for the implementation and operationalization of surveillance systems for diseases of viral origin [5]. In this context, the World Health Organization (WHO) has established strategies to reduce the impact of seasonal influenza, strengthening prevention and control measures for animal-to-human transmission by 2030 [6]. Due to the underreporting of cases in Mozambique during the 2009 pandemic, a collaboration between the Ministry of Health (MoH) and the National Institute of Health (NHI) was established in 2013 to implement an influenza surveillance system based on sentinel posts (SP). In 2020, influenza monitoring and response in Mozambique was affected by COVID-19. In this context, it is intended to evaluate the performance of the influenza sentinel surveillance system.

## Methods

The evaluation was carried out in Maputo City, in the period between January 2016 and December 2021. The study population was considered, individuals of all ages, living in the areas covered by the study site. Suspected cases were individuals with epidemiological and clinical history of fever ≥38°C and cough for more than three days, with at least two of the following symptoms: shortness of breath, runny nose, odynophagia, and headache started within 10 days. The cases were confirmed with positive polymerase chain reaction (PCR) test results. The influenza laboratory database and patient records of the Maputo Central Hospital (MCH), Mavalane General Hospital (MGH), Mavalane Health Center (MHC) obtained from the NHI virology laboratory were the sources of the data. In an approximate universe of 5,000 records of patients seen in the period under study and considering the formula of Levin;


$$\frac{N}{\left(1+N*e^{2}\right)}$$

where N represents the population size, corresponding to the estimate total of 5,000 suspected cases; e denotes the estimated sampling error et set at 5% (α=0.05); and n is the calculated sample size. Based on this calculation, a sample of 372 files was obtained, which 62 records were evaluated per year. Subsequently, a simple random sampling technique was applied to select the registration forms for assessing data quality. The description of the influenza sentinel surveillance system was elaborated based on the review of the protocols and documents. The attributes were evaluated through the script of the Centers for Disease Control and Prevention of the USA (2001) [7]. The classification and scoring of the attributes were based on the articles of the countries with incidence of influenza [8,9].

**Data management and analysis:** Microsoft Excel 2019 and Tableau 2018.1 Software were used for data cleansing and analysis. The ArcMap 10.2.2 tool was used to prepare a distribution map of the cases. The study variables were “date of collection and reception of the sample”, “onset of symptoms”, “PCR result”, “strain”, “provenance”, “sex” and “age”. The ethical conditions of this evaluation are in line with the objectives of the protocol for influenza surveillance approved by the National Committee for Bioethics in Health, whose reference is IRB: 00002657.

**Figure 1:**
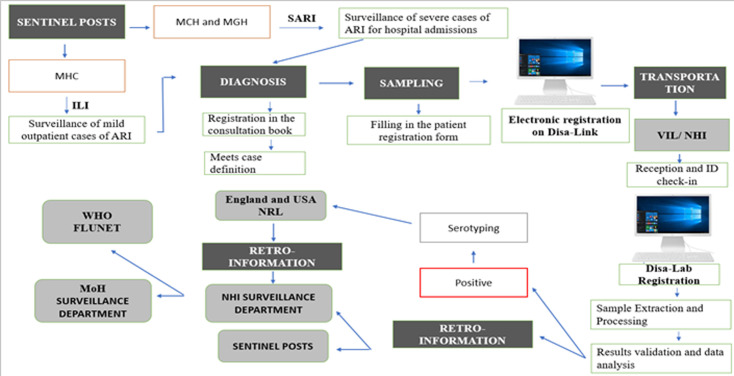
flowchart of operation of influenza surveillance system

**Description of the system:** the influenza surveillance system contains a physical and electronic form for data collection. At the consultation, if the patient has symptoms suggestive of influenza is recorded in the physical and electronic form Disa-Link. After collection, the samples are organized and sent with the registration forms for testing and validation in the virology laboratory of the NHI. The results are recorded in the Disa-Lab registration form and the electronic results form to be viewed in the SP. Subsequently, registration form data without patient identification data are entered into the influenza laboratory database and archived. The report is prepared and shared weekly with the NHI Surveillance Department. Finally, the data are entered into the WHO FluNet Platform (Figure 1).

### Evaluation of attributes

**Data quality:** the completeness and consistency of the data recorded in the database and registration forms were assessed. Rating: good >85%, regular 50-80%, and bad <50%.

**Stability:** it was considered whether the system remained operational during the study period. Rating: stable >70%; regular 50-70%; not stable <50%.

**Sensitivity:** the positivity rate of the samples was verified during the study period.

**Representativeness:** it was based on the analysis of the distribution of cases by neighborhoods in the area covered by the Maputo SP between 2016-2022. Classification: non-representative <50%, medium: 50-80%, representative >80%.

**Opportunity:** it was based on the verification of the laboratory response time (LRT) from the collection of the sample in the SP to the processing during the study period. Classification: timely ≥80% and non-timely <80%, Table 1.

**Positive predictive value (PPV):** it was verified whether the system demonstrates accuracy in the detection of cases that meet the case definition. In the end, the performance of the system was considered through the results of each attribute evaluated to verify if it meets the recommended objectives (Table 1).

## Results

**Evaluated attributes:** of the 4,819 samples received in the last six years, 96.7% (4,660/4,819) were processed and recorded in the influenza laboratory database. Fifty-four percent ((54%) 2,617/4,660) of the samples analyzed were male.

**Data quality:** the system obtained a completeness of 69.9% (3,260/4,660). The variables date of “collection” and “reception” of the sample had 100% and 85.3% (3,976/4,660) of completion. Data consistency between database and record form was 68% (355/372). The variable “strain” presented a mean difference of 5% of consistency between the form and the database. The system presented an average data quality of 68.9% (Table 1).

**Table 1:** data consistency between the laboratory database and patient record form, 2016-2021

Year	Date of onset of symptoms			Date of sampling			PCR results			Strain specification		
	DB	RF	C (%)	DB	RF	C (%)	DB	RF	C (%)	DB	RF	C (%)
	*N (%)	**n (%)		N (%)	n (%)		N (%)	n (%)		N (%)	n (%)	
2016	1,562 (98.7)	34 (54.8)	56.1	1,582 (100)	62 (100)	100	1,400 (88.4)	47 (75.8)	87.4	46 (2.9)	3 (4.8)	96.1
2017	1,052 (98.0)	40 (64.5)	66.5	1,068 (100)	42 (67.7)	67.6	958 (90.0)	50 (80.6)	90.6	92 (8.6)	7 (11.2)	97.4
2018	1,164 (96.0)	52 (83.8)	87.8	1,202 (100)	57 (91.9)	91.9	1,096 (91.1)	62 (100)	91.1	16 (1.3)	18 (29.0)	8.6
2019	439(95.0)	54 (87.0)	92.0	452 (100)	58 (93.5)	93.5	378 (84.0)	60 (96.7)	87.3	12 (3.0)	4 (6.4)	36.1
2020	7 (100)	60 (96.7)	96.7	7(100)	58 (93.5)	93.5	0 (0)	41 (66.1)	0	0 (0)	0 (0)	0
2021	339 (97.1)	44 (71.0)	73.9	349 (100)	58 (94.0)	94	246 (70.4)	46 (74.1)	96.0	0 (0)	0 (0)	0
**Total**	4,583 (98.3)	284 (76.3)	78.0	4,660 (100)	335 (90)	90	4,078 (88.0)	306 (82.2)	94.2	166 (4.0)	32 (9.0)	38.4

**DB:** Database; **RF:** Registration forms; **C:** Consistency; PCR: polymerase chain reaction * Denominator/year: 2016 (N=1,582), 2017 (1,068), 2018 (N=1,202), 2019 (N=452), 2020 (N=7), 2021 (N=349); N= 4,660 ** sample: 62/year evaluated; n=372

**Stability:** regarding sample processing, 96.7% (4,660/4,819) of the samples were processed between 2016-2021, being 93.9% (1,582/1,684) in 2016, 98.3% (1,068/1,086) 2017, 99.8% (1,202/1,205) 2018, 97.6% (452/463) 2019, 0.6% (7/1,264) in 2020 and 38.7% (349/901) in 2021 (Table 2).

**Table 2:** results and classification of influenza virus surveillance system evaluation attributes

attribute	evaluation method	parameter	punctuation	results	classification
Data quality	Completeness: number of essential variables filled in the database: “provenance”, “sex”, “age”, “type of sample”, “date of sampling” and “date of receipt of the sample”	# of (...) fields filled in per year x100% Total fields completed per year	Classification (x̄=ʃ/n): 0 to 100% Good (>85%) Regular= (50-85%) Poor (<50%)	Completeness =69.9% Consistency = 68%	Regular data quality
	Consistency: % of the variable “date of onset of symptoms”, “date of sample collection”, “PCR result” “strain” filled in between patient records and SARI database	Quantity of variable (..) concordant between Sheet and database X100% Total variables filled			
Stability	System functionality: annual temporal distribution of samples received and processed between 2016-2021	# samples analyzed X 100% Total samples received from suspected cases	Classification: 0 to 100%/year Stable: >70% Regular: 50 to 70% Not stable <50%	>80% reduction in laboratory testing capacity between 2020 and 2021 2016: 93.9% (1,582/1,684) 2017: 98.3% (1,068/1,086) 2018: 99.8% (1,202/1,205) 2019: 97.6% (452/463) 2020: 0.6% (7/1,264) 2021: 38.7% (349/901)	Stable
Sensitivity	Positivity rate: total positive (confirmed) cases in the year of highest notification of Cases (2017)	# confirmed cases w/disease X 100% Total samples processed from suspected cases	Classification: 0 a 100%/year High: ≥70% Low: <70%	Positivity rate general of 28% (1,305/4.660) 2016: 3.3% (52/1,582) 2017: 77.5% (842/1,086) 2018: 32.8 (394/1,202) 2019: 3.8% (17/452) 2020: 0% (0/7) 2021: 0% (0/349)	Not sensitive
Representativeness	Geospatial distribution of suspected and confirmed cases by neighborhood in Maputo city, 2016-2021	# cases with laboratory diagnosis confirmed by provenance X 100% Total samples processed from suspected cases	Classification (x̄=Ʃ/n): 0 a 100% Representative: ≥80% Non-representative: <80%	Representativeness= 98.4%	Representative
Timeliness	Laboratory response time	Date of issue of result Date of receipt of sample ≤7 days = 1 point >7 days = 0 points	Score: 0 to 1 point/item Classification: 0 to 100% Timely: ≥80% Non-timely: <80%	LRT in 7 days = 50.4%	Not timely
Predictive positive value (PPV)	Percentage of suspected cases that met the case definition that have been tested and reported in the surveillance system	# suspects met case definition X 100% Total confirmed cases (a/a+b)	Classification (x̄=Ʃ/n): 0 a 100 % High: ≥50% Low: <50%	PPV = 31.4%	**Low PPV**

**Sensitivity:** during the study period, the overall positivity rate was 28% (1,305/4,660), being 3.3% (52/1,582) in 2016, 77.5% (842/1,086) in 2017, 32.8 (394/1,202) in 2018, 3.8% (17/452) in 2019, 0% (0/7) and (0/349) in 2020 and 2021 respectively (Table 2).

**Representativeness:** ninety-eight point four (98.4%) (1,285/1,305) of the neighborhoods covering the SP of Maputo had at least one positive case among the suspected cases reported (Table 2).

**Opportunity:** fifty point four (50.4%) (2,349/4,660) were processed within seven days from the date of receipt (Table 2).

**Positive predictive value (PPV):** thirty-one point four (31.4%) (410/1,305) of the cases reported in the system meet the case definition, and yet are considered the true positives (Table 2).

**Utility:** about 50% (3/6) of the objectives recommended by the sentinel influenza surveillance system for describing the epidemiological situation and detecting cases were achieved during the evaluation of the system's performance.

## Discussion

Easy-to-manage, stable, timely, and low-cost epidemiological surveillance systems are preferred. The sentinel system implemented for detecting outbreaks and predicting influenza waves had <80% of samples tested in time between 2020-2021. This can be explained by the use of this system to respond to the COVID-19 emergency, and yet making it impossible to monitor influenza viral co-circulation during the pandemic due to system instability. This, however, influences the detection of influenza cases in the population under surveillance and, therefore, the prediction of new waves. A study on the impact of COVID-19 on tuberculosis detection in Mozambique showed a 15% reduction between expected and reported cases in the system in the same period [10]. Important measures should be taken to improve the operationalization of the system and ensure national expansion to prevent re-emergencies of potential pandemics and epidemic agents associated with human-animal interaction.

**Limitations:** due to its inoperability, it was not possible to evaluate the influenza sentinel surveillance system at the reference sentinel posts in the central and northern regions during the study period, which is why the evaluation was limited in the city and province of Maputo.

## Conclusion

The influenza sentinel surveillance system is structured, with well-defined levels of responsibility and instruments capable of responding with reliable data. The system can describe the seasonality of the influenza virus and estimate basic rates for detecting cases in the population. Efforts to identify the types and subtypes of the circulating influenza virus and their relationship to global standards should be improved to predict occurrences over time. The use of more specific criteria for the diagnosis and collection of influenza virus samples in the PS and the reduction of laboratory response time would contribute to strengthening the capacity of the system, with results higher than 80% in all attributes evaluated, ensuring adequate monitoring and prevention of potential respiratory epidemics.

## References

[1] Schmidt SSS, Luliano AD, Vestergaard LS, Mazagatos-Ateca C, Larrauri A, Brauner JM (2022). All-cause versus cause-specific excess deaths for estimating influenza-associated mortality in Denmark, Spain, and the United States. Influenza Other Respir Viruses.

[2] Ministério da Saúde de Moçambique (2019). Gripe sazonal.

[3] Rabarison J, Tempia S, Harimanana A, Guillebaud J, Razanajatovo NH, Ratsitorahina M (2019). Burden and epidemiology of influenza-and respiratory syncytial virus-associated severe acute respiratory illness hospitalization in Madagascar, 2011-2016. Influenza Other Respir Viruses.

[4] Siqueira GA (2013). Influenza a (h1n1) 2009 epidemic in the state of Goias/Brazil: cases and deaths. (*Epidemia da Influenza A (H1N1) 2009 no Estado de Goiás/Brasil: Casos e Óbitos*).

[5] Mazalo J, Mori B, Boechat AL (2021). Challenges faced in implementing prevention measures to contain the novel coronavirus in Africa: a review of measures adopted in South Africa, Algeria and Nigeria (*Desafios Enfrentados na Implementação das Medidas de Prevenção para Conter o Novo Coronavírus em África: uma Revisão sobre Medidas Adotadas na África do Sul, Argélia e Nigéria*). Revista Desafios.

[6] Biblioteca Virtual em Saúde WHO launches new global strategy to control influenza (flu). (*A Organização Mundial da Saúde Lança Nova Estratégia Mundial Para Controle da Influenza (Gripe)*.

[7] German RR, Horan JM, Lee LM, Milstein B, Pertowski CA (2001). Updated guidelines for evaluating public health surveillance systems: recommendations from the Guidelines Working Group. MMWR Recomm Re.

[8] Ribeiro Igor, Sanchez MN (2020). Evaluation of the severe acute respiratory syndrome (SARS) surveillance system, with emphasis on influenza, Brazil, 2014-2016. Epidemiol Serv Saude.

[9] Biblioteca Digital Brasileira de Teses e Dissertações (2010-2013). Evaluation of the influenza epidemiological surveillance system in Brazil 2010-2013. *Avaliação do sistema de vigilância epidemiológica da influenza no Brasil*.

[10] Manhiça I, Augusto O, Sherr K, Cowan J, Cuco RM, Agostinho S (2022). COVID-19-related healthcare impacts: an uncontrolled, segmented time-series analysis of tuberculosis diagnosis services in Mozambique, 2017-2020. BMJ Glob Health.

